# Person-centered maternity care and associated factors among women who give birth at public hospitals in South Gondar zone, North West Ethiopia, 2023

**DOI:** 10.1371/journal.pone.0303389

**Published:** 2024-08-22

**Authors:** Gizachew Worku Dagnaw, Mekonen Melkie Bizuneh, Meseret Birhan Kassie

**Affiliations:** Department of Reproductive Health and Population Studies, School of Public Health, College of Medicine and Health Science, Bahir Dar University, Bahir Dar, Ethiopia; Addis Ababa University, ETHIOPIA

## Abstract

**Background:**

Person-centered maternity care (PCMC) is the process of maternity care that is responsive to and respectful of each woman’s choices, values, and needs. It reflects the quality of maternal health services. The provision of PCMC is influenced by the maternal sociodemographic and obstetric-related variables However, there is little information about person-centered maternity care in Ethiopia; particularly, communication & autonomy, and supportive care are not well investigated. Therefore, the purpose of this study was to assess the proportion of person-centered maternity care and associated factors among women who give birth at the public hospital, in North West, Ethiopia, 2023.

**Methods:**

A facility-based cross-sectional study was conducted among 763 mothers who give birth at public hospitals in the South Gondar zone. The study participants were selected using a systematic random sampling technique. Data were collected through exit interviews using a structured pretested Amharic version questionnaire. EPI- Data version 4.6 was used for data entry and SPSS Version 25 for analysis. Bivariable & multivariable linear regression analysis was computed. Predictor variables were identified by using unstandardized β and a 95% confidence interval. A variable that has a p-value < 0.05 was considered statistically significant.

**Results:**

The mean person-centered maternity care was 42.33 out of 90. Mothers living in rural areas (β = -1.43, 95% CI: -2.76,-0.11), 3–4 providers present during labor and delivery (β = -1.58, 95% CI:-3.67,-0.27), had no history of facility delivery (β = -2.28,95% CI:-4.43,-0.13), two days length of stay at hospitals (β = 1.69,95% CI:0.40,2.48), and highest wealth (β = 1.05,95% CI:0.42,1.41) were factors significantly associated with person-centered maternity care (PCMC).

**Conclusion:**

The mean score of PCMC was low in the study area compared to studies done in low- and middle-income countries. Highest wealth, 3–4 health providers present during labor and delivery, had no history of facility delivery, and had two-day lengths of stay at hospitals were predictors for PCMC. Therefore, strengthening facility delivery and promoting hospital stays for more than a day as a part of first postnatal care is paramount for increasing PCMC.

## Introduction

Person-centered maternity care (PCMC) is the process of maternity care that is responsive to and respectful of each woman’s choices, values, and needs; and it considers when making clinical decisions during labor and delivery [1]. The World Health Organization (WHO) identified three key PCMC components that all mothers should get throughout labor and delivery: dignity and respect, communication, and supportive care [2, 3]. Person-centered care is recognized as a crucial component of the quality of maternity care that takes into consideration user experience and influences health-seeking behavior throughout childbirth [2]. Providing PCMC can help in better patient-provider communication, more treatment adherence, timely care delivery, and better maternal and neonatal outcomes [4].

High Maternal mortality rates, particularly in sub-Saharan Africa, are a result of poor healthcare standards. About three-quarters of maternal deaths result from complications during childbirth and the first 24 hours following delivery [5]. The PCMC score in low- and low-middle-income nations were low; ranging from 51.6% in rural Ghana to 66.9% in urban Kenya. Moreover, 90% of health professionals didn’t introduce themselves to the mother [4].

PCMC was affected by the mother’s residence, time of delivery, mode of delivery, and length of stay in health facilities [4, 6, 7]. Advancing PCMC approaches for maternal health services is essential to promote facility delivery and satisfaction with service and ensure the implementation of women’s rights [8]. Efforts to encourage the use of maternal health services in low- and middle-income nations are not possible to achieve the desired goals without enhancing women’s experience with quality care. Studies revealed that women prefer to give birth in settings that make them feel valued, safe, and respected [3, 9, 10].

Different global and national strategies and interventions are undertaken to promote the provision of PCMC [11–14]. Providing women-centered maternity care is one of the global approaches identified to improve the health of the mother and reduce death-related pregnancy and delivery [2, 15]. PCMC is vital for delivering the subsequent maternal and child health service packages. Women with high PCMC scores expressed a greater readiness to deliver future children at the healthcare facility than did women with lower PCMC scores [8, 16]. On the other hand, Poor healthcare experiences could be an obstacle for women and children seeking healthcare services; weakened deliveries and experience of complications are strongly linked to poor PCMC [4, 17, 18]. Despite this fact, there is little evidence on some dimensions of PCMC to identify the real client’s experience of PCMC [19, 20]. Particularly, communication, autonomy, and supportive care are not well researched in Ethiopia in general and in the South Gondar zone in particular. As a result, the goal of this study was to assess the PCMC and associated factors among women who give birth at public hospitals in the South Gondar zone, Amhara region, North West Ethiopia.

## Methods and materials

### Study design and period

A facility-based cross-sectional study was conducted among women who gave birth in South Gondar zone public hospitals from March 20, 2023- May 21, 2023. South Gondar zone is among 15 administrative zones in the Amhara region. Debre Tabor is the capital town of the zone which is located 669 Km northwest of Addis Ababa, the capital city of Ethiopia, and 97 Km southeast of Bahir Dar, the capital city of the Amhara region. Available information from the zonal health office shows that the South Gondar zone has an estimated population of 2,696,297, of which 50.2% (1,353,541) were women. South Gondar zone has one referral hospital and nine primary hospitals that deliver maternal and child health services. According to the zone health department report 2022/2023, in such health facilities an estimated number of 90,865, 69,057&72,692 mothers received antenatal care, delivery, and postnatal care services respectively.

### Study population and eligibility criteria

All women who gave birth in randomly selected public hospitals during the data collection period were included in this study. Women who were referred from other health institutions to those selected public hospitals after giving birth were excluded from the study.

### Sample size determination and sampling procedure

The sample size was determined using a single population proportion formula. Considered the following assumption; proportion of PCMC; 64.5% [6], standard normal distribution at a 95% confidence level, 5% margin of error, and design effect of two. Based on this assumption the calculated sample size was 704. Then after considering a 10% possible non-response rate, the final sample size was 774.

Five public hospitals were selected out of ten governmental hospitals found in the South Gondar zone using a lottery method. These were Debre Tabor referral hospital, Andabet primary hospital, Addis Zemen primary hospital, Mekane Eyesus hospital, and Wogeda primary hospital. The total sample size (774) was distributed proportionally to each of the selected hospitals based on the previous year’s similar two-month delivery report on each hospital. The study participants were recruited using a systematic random sampling technique. The sampling interval (K) was determined using the previous year’s similar two-month client load (N) from the five selected health facilities divided by the calculated sample size (n) (K = N/n = 1904/774 = 2.45). So, every two mothers were interviewed. The first mother was selected randomly by lottery method and then continued based on the sampling interval (S1 Fig).

### Study variables

#### Dependent variable

Person-centered maternity care

#### Independent variables

- Socio-demographic variables: age, residence, level of education, employment status, monthly income (wealth).
- Obstetrics-related variables: ANC, mode of delivery, time of delivery, complication during delivery, index of birth outcome, and previous delivery experience.
- Facility and health service-related variables: the number of providers who care for the mother, sex of the health professional, profession of delivery attendant, and length of stay at a health facility.

### Operational definition and its measurement

#### Person-centered maternity care

Person-centered maternity care was measured by using the PCMC scale. The PCMC scale has three domains: communication and autonomy (9 items), dignity and respect (6 items), and supportive care (15 items) with a total of 30 items; each item having a four-point response scale. i.e., 0 = ‘no, never’, 1 = ‘yes, a few times, 2 = ’yes, most of the time ’, and 3 = ’yes, all the time’. During the data entry, the negatively worded items were reversely coded so that the highest score represents good care and allowed all data to be combined and statistically analysed with the positively worded item questions. The total PCMC score was a summative score from the response to individual items which ranges from 0 to 90 [6, 7, 21].

#### Length of stay

The number of days the mother stays in the hospital after giving birth, was recorded at the time of client discharge.

### Data collection tool and procedures

Data were collected using a structured pretested Amharic version questionnaire. First, the English version questionnaire was adopted from different sources. The adopted tool was validated in different low—and middle-income countries to assess person-centered maternity care that had good internal reliability with Cronbach’s alpha above 0.8 [4, 22, 23]. Then the English version questionnaire was translated into the local language (Amharic) and retranslated back into English language to check its consistency.

The questionnaires comprise socio-demographic characteristics of the mother, obstetrics history, facility and health service characteristics, and person-centered maternity care scale questionnaires. Data were collected through face-to-face exit interviews using a structured questionnaire and document review using a checklist. The profession of delivery attendant, mode of delivery, and complications during delivery were collected from the client chart. Five diploma data collectors and two BSc midwife supervisors participated in the data collection. To reduce the introduction of courtesy bias both data collectors and supervisors were recruited out of the study setting. Moreover, the principal investigator managed the whole process of the data collection procedure.

### Data quality assurance

To get more accurate information, data collection was done using the participant’s mother tongue language (Amharic). The one-day training was given to supervisors and data collectors on the roles and responsibilities as well as the overall data collection procedure. Additionally, a pretest was done on 5% of the sample size (39 women) at Debre Tabor referral hospital before the actual data collection period to check whether the questionnaires were simple, clear, and easily understandable. Based on the pre-test findings necessary modifications of sequence and word were made. The investigator and supervisors checked the data each day to make sure it was accurate and consistent.

### Data processing and analysis

The collected data was checked for consistency and completeness by the principal investigator. Then, the data were cleaned, coded, and entered into EPI- Data 4.6 and exported into SPSS Version 25 for analysis. Both descriptive and analytical statistics were done and presented using text, figures, and tables. After creating dummy variables simple linear logistic regression analysis was conducted to select candidate variables for multiple linear logistics regression. The assumption of linearity was checked by scatter plot and normality was checked by using Q-Q plots and histogram. The multi-collinearity assumption was checked by a variance inflation factor (VIF) with a maximum value of 3.7 that confirms no multi-collinearity, The Durbin-Watson statistic was used to check the assumptions that the residual values are independent with a Durbin-Watson statistics value of 1.53. The model fitness was checked by multiple correlation coefficients and ANOVA test significance. Variables having p-value ≤ 0.25 in simple linear logistic regression were entered into multiple linear logistic regressions for controlling the possible effect of confounders. Predictor variables were identified using unstandardized β and 95% CI. Variables that have a p-value < 0.05 were considered statistically significant.

### Ethical approval statement

Ethical clearance was obtained from the Bahir Dar University College of Medicine and Health Sciences Ethical Review Board (ERB) with an ethical reference number (CMHS/IRB: 701/2023). Then support letters were received from Amhara Public Health Institute (APHI). A participant was informed about the objective of the study before data collection and informed written consent was taken from all study participants. For participants aged below 18 years written consent was taken from their legal guardians. A voluntary participant was informed that they have the right to refuse or withdraw whenever they want at any stage of data collection. Confidentiality was maintained by omitting names and personal identifiers throughout the study.

## Results

### Socio-demographic characteristics of participants

Seven hundred sixty-three mothers have participated in the survey with a response rate of 98.5%. Most of the study participants, 328 (71.6%) were living in rural areas. The mean age of the participants was 29 ± 5.05 years. About 319 (41.8%) of the study participants did not attend formal education. Regarding occupation 481 (63%) were farmers and 192 (25.2%) mothers had the lowest wealth quintiles (Table 1).

**Table 1:** Socio-demographic characteristics of the participants at public hospitals in South Gondar zone, Ethiopia, 2023 (n = 763).

Variables	Category	Frequency	Percentage (%)
Residence	Rural	549	71.6
	Urban	214	28.4
Age of the mother	16–20	24	3.1
	21–30	454	59.5
	31–40	273	35.8
	>40	12	1.6
Mother education	No formal education	319	41.8
	Primary education (1–8)	170	22.3
	Secondary education (9–12)	88	11.5
	Higher education	181	24.3
Mother’s Occupation	Farmer	481	63
	Government employee	139	18.2
	Merchant	75	9.8
	Private employee	43	5.6
	Student	25	3.3
Wealth quantile	Lowest	192	25.2
	Second	109	14.3
	Middle	156	20.4
	Fourth	168	22.0
	Highest	138	18.1

### Obstetrics, facility, and service-related characteristics of the respondents

Of the total 763 study participants, all 763 (100%) had antenatal care follow-up for the index delivery. Of 275 (36%) mothers had received four and above ANC visits. More than half (54.5%) of mothers had delivered through spontaneous vaginal delivery and 670 (87.7%) of labor was attended by midwife nurses. Out of ten, nine (89.8%) of mothers’ labor was attended by three and above health providers. Similarly, 601 (78.8%) deliveries were attended by both male and female providers. Nearly half (50.9%) of mothers had given birth in the daytime. The majority of participants, 633 (83%) had no complications during delivery. Regarding the birth outcome, 35(4.6%) had dead newborn outcomes. Among the total respondents, 564 (73%) had only one-day length of stay at hospitals (Table 2).

**Table 2:** Obstetrics, facility & health service -related characteristics of the participants at public hospitals in South Gondar zone, Ethiopia, 2023 (n = 763).

Variables	Category	Frequency	Percentage (%)
Number of ANC visits for the index birth	Less than four	488	64
	Four and above	275	36
Number of previous live birth	Less than four	663	86.9
	Four and above	100	13.1
History of previous facility delivery	No	740	97
	Yes	23	3
Mode of delivery of the index birth	Spontaneous vaginal delivery	416	54.5
	Instrumental	250	32.8
	Cesarean section	97	12.7
Time of delivery of the index birth	Day	388	50.9
	Night	375	49.1
Complication during delivery	No for both	633	83
	Yes, for mother	75	9.8
	Yes, for neonate	55	7.2
The outcome of the index birth	Alive	728	95.4
	Died	35	4.6
Number of health providers present during labor & delivery	1–2	78	10.2
	3–4	602	78.9
	**> = 5**	83	10.9
Sex of delivery attendant	Both male and female	601	78.8
	Male	116	15.2
	Female	46	6
Length of stay at hospitals	One day	564	73.9
	Two days	128	16.8
	More than two to one week	71	9.3
Profession of delivery attendant	Midwife	670	87.8
	Doctor	47	6.2
	Emergency surgeon	46	6

### Person-centered maternity care (PCMC) scales

The participants’ mean PCMC score was 42.3 (±5.2 SD) with minimum and maximum scores of 20 and 63 out of 90 respectively. About 47% (95% CI; 43–50) of the respondents scored more than the mean PCMC score. The mean PCMC score for sub-scales was 13.71 out of 18 for dignity and respect, 4.89 out of 27 for communication and autonomy, and 23.72 out of 45 for supportive care.

#### Dignity and respect

The participants’ mean dignity and respect scores were 13.71 ± 1.2 SD out of 18. The majority, 731 (95.8%) of respondents reported that that their interactions with healthcare professionals were respectful. Around half, 386 (50.6%) of the respondents reported health providers treated them in an unfriendly manner. Fourteen (1.8%) clients claimed that their auditory privacy was violated. Similarly, 17 (2.2%) clients believed their health information was not maintained confidentially (Table 3).

**Table 3:** Dignity and respect, communication and autonomy and supportive care items in public hospitals, South Gondar zone, Ethiopia, 2023 (n = 763).

Items	No, never (%)	Yes, a few times (%)	Yes, most of the time (%)	Yes, all the time (%)
**Dignity and Respect**				
Providers treat with respect	10(1.3)	15(2.0)	731(95.8)	7(0.9)
Providers treat in a friendly manner	386(50.6)	18(2.4)	355(46.5)	4(0.5)
Providers shouted, insulted, scolded, threatened, or told me rudely **(RC)**	667(87.4)	88(11.5)	8(1.0)	0
Providers pushed, slapped, beaten, pinched, or physically restrained **(RC)**	759(99.5)	0	4(.5)	0
People not involved in the care hear discussions with the provider **(RC)**	748(98)	1(.1)	13(1.7)	1(0.1)
Confidentiality is protected during a hospital stay	17(2.2)	6(.8)	729(95.5)	1(0.1)
**Communication and Autonomy**				
Providers introduced themselves	751(98.4)	2(.3)	10(1.3)	0
Providers call me by my name	647(84.8)	10(1.8)	103(13.5)	3(0.4)
Involved in decisions	699 (91.6)	4 (.5)	60 (7.9)	0
Consent before doing examinations and procedures	693 (90.8)	6 (.8)	64 (8.4)	0
Allowed position of choice	718 (94.1)	7 (.9)	38 (5.0)	0
Providers speak in a language	40 (5.2)	1 (.1)	70 (9.2)	652 (85.5)
The purpose of examinations and procedures was explained	667 (87.4)	11 (1.4)	81 (10.6)	4 (.5)
The purpose of medicines explained	497 (65.1)	12 (1.6)	252 (33)	2 (.3)
Could ask any questions	592 (77.6)	167 (21.9)	4 (.5)	4 (0.5)
**Supportive Care**				
Allowed a labor companion	517(67.8)	1 (.1)	243 (31.8)	2 (0.3)
Allowed a delivery companion	752 (98.6)	11 (1.4)	0	0
Talk to you about how you were feeling	232 (30.4)	21 (2.8)	509 (66.7)	1 (0.1)
Providers supported anxieties and fears	10(1.3)	217 (28.4)	528 (69.2)	8 (1.0)
Providers try to manage pain	9 (1.2)	227 (29.8)	520 (68.2)	7 (0.9)
Providers in the facility paid attention	10 (1.3)	234 (30.7)	516 (67.6)	3 (0.4)
Providers took the best care	7 (.9)	226 (29.6)	525 (68.8)	5 (0.7)
Completely trust the providers in the facility about care	5 (.7)	224 (29.4)	530 (69.5)	4 (0.5)
There were enough providers	9 (1.2)	24 (3.1)	724 (94.9)	6 (0.6)
The rooms were crowded **(RC)**	725 (95)	22 (2.9)	15 (2)	1(0.1)
There was water in the facility	52 (6.8)	391 (51.2)	319(41.8)	1 (0.1)
There was electricity in the facility	16 (2.1)	145 (19)	602(78.9)	0
Overall safety during hospital stays	3 (.4)	107 (14)	649(85.1)	4 (0.5)
Waiting time	Very long 5 (.7)	Somewhat long 81 (10.6)	Little long 647 (84.8)	Very short 30 (3.9)
The general environment of the facility	Very dirty 4 (.5)	Dirty 410 (53.7)	Clean 348 (45.6)	Very clean 1 (0.1)

RC- reverses coding

#### Communication and autonomy

Regarding the autonomy and communication scale, the participants scored a mean of 4.89 ±2.9SD out of 27. Seven hundred fifty-one (98.4%) participants said that providers never introduced themselves while providing health care to them. More than three-quarters, 647 (84.8%) said that healthcare providers never call them using their names. Six hundred ninety-three respondents (90.8%) reported that healthcare professionals never sought consent before performing physical examinations and procedures. Similarly, 718 (94.1%) respondents reported that health providers never allowed them to choose their own preferred delivery positions. Around two-thirds (65.1%) of the respondents stated that their healthcare professional failed to explain the reason for the prescribed medication. Only sixty respondents (7.9%) participated in decision-making regarding their care (Table 3).

#### Supportive care

In this study, the participants’ mean score on the supportive care scale was 23.72 ± 3.2SD out of 45. The majority, 752 (98.6%) of the respondents reported that they were prohibited from having companions at the time of delivery. However, 724 (94.9%) respondents reported that there were adequate healthcare providers to take care of them. Besides, 725 (95%) of the mothers reported that they never experienced overcrowding in the rooms while they were staying at the facility. Moreover, 649 (85.1%) respondents said that they felt comfortable there (Table 3).

### Factors associated with person-centered maternity care

Using simple linear logistic regression analysis mother’s occupation, number of antenatal care services, mode of the index birth, newborn birth outcome, number of providers present during labor and delivery, the profession of delivery attendant, previous history of facility delivery, residence, length of stay at hospitals, and wealth index were a candidate for multiple linear regression.

After controlling possible confounders using multiple linear logistic regression analysis; having the highest wealth index, three up to four providers present during labor delivery, had no previous history of facility delivery, rural residence, and had a two-day hospital stay for the index birth were factors significantly associated with PCMC score.

Keeping other variables constant, mothers living in rural areas had decreased person-centered maternity care scores by 1.43 as compared to mothers living in urban areas (β = -1.43, 95% CI: -2.76, -0.11). Keeping other variables constant, when three up to four health providers were present during labor and delivery the person-centered maternity care score was decreased by 1.58 as compared to one up to two providers present (β = -1.58, 95% CI: -3.67, -0.27). Keeping other variables constant, mothers who had no previous history of facility delivery had decreased person-centered maternity care scores by a factor of two as compared to their counterparts (β = -2.28, 95% CI: -4.43, -0.13).

Keeping the other variable constant, mothers who stay at hospitals for two days increase the person-centered maternity care score by 1.69 as compared to mothers who stay at hospitals for one day (β = 1.69, 95% CI: 0.40, 2.48). Furthermore, mothers in the highest wealth quintiles had increased PCMC scores by one as compared to mothers in the lowest wealth quintiles (β = 1.05, 95% CI: 0.42, 1.41) (Table 4).

**Table 4:** Bivariable and multivariable linear logistic regression analysis for factors affecting person-centered maternity care in public hospitals, South Gondar zone, Ethiopia, 2023.

Variables	Category	Unstandardized B coefficient	95%CI
Mother Occupation	Student	1.00	
	Farmer	-0.48	(-1.20, 2.00)
	Merchant	1.65	(0.42, 2.89)
	Private employee	-0.59	(-2.20,1.00)
	Government employee	-0.43	(-1.39, 0.52)
Residence	Urban	1.00	
	Rural	-1.65	(-3.04, -0.20) *
Wealth	Lowest	1.00	
	Second	0.07	(-1.10, 1.24)
	Middle	0.49	(-0.42, 1.41)
	Fourth	0.58	(-0.39, 1.55)
	Highest	1.05	(0.42, 1.41) **
Number of ANC visits for the index birth	Four and above	1.00	
	Less than four	-0.56	(-1.33, 0.20)
Previous history of facility delivery	Yes	1.00	
	No	-2.28	(-4.43, -0.13) *
Mode of delivery of the index birth	CS	1.00	
	Instrumental	-0.86	(-1.09, 0,50)
	SVD	0.34	(-0.39, 1.08)
newborn birth outcome	Dead	1.00	
	Alive	-1.38	(-1.76, 2.00)
Number of health providers present during labor& delivery	1–2	1.00	
	3–4	-1.58	(-2.67, -0.27) *
	>5	1.23	(-3.08, 0.38)
Length of stay at hospitals	1day	1.00	
	2day	1.69	(0.40, 2.48) **
	2-1wk	1.88	(-0.26, 2.80)
Profession of delivery attendant	Emergency surgeon	1.00	
	Doctor	1.73	(-1.31, 2.12)
	Midwife	-1.09	(-2.22, 0.03)

Keys: 1 = Reference, CI = Confidence Interval

* = p- value< 0.05

** = p- value< 0.01

*** = p- value< 0.001

## Discussion

This study investigated the score of PCMC and its determinants among mothers who gave birth in public hospitals in the South Gondar zone, northwest Ethiopia.

In this study, the mean score of PCMC was 42.3(±5.2 SD). The lowest score was on the communication & autonomy subscale, while the highest was on the respect and dignity dimension. The finding of this study is consistent with the studies done in Colombo and Siri Lanka (42.3 out of a maximum score of 90) [8] and a systemic review and meta-analysis study done in Sub-Saharan Africa (44%) [24]. However, it was lower than a study conducted in Addis Ababa Ethiopia (59.2 out of 90) [7], Nairobi Kenya (58.2 out of 90) [25], and a study done among three low and middle-income countries (Rural Kenya, Urban Kenya, and India with a PCMC score of 59.5, 60.2, and 55,8out of 90 respectively) [4]. The possible reason for this difference could be due to socioeconomic differences (income, education, wealth). In the current study, 25% of mothers have the lowest wealth quantile and 41.8% of the respondents had no formal education. Whereas, in a study done in Addis Ababa more than 90% of the respondents attended primary education and above and 38.6% of the participants also had the highest wealth quantile [7]. The other reason for the higher PCMC score in the aforementioned studies might also be the difference in the study setting; more than 70% of the study participants in the current study were rural residents and the equivalent figure for the above two studies was below 10% [4, 7]. Urban women have better education opportunities, health information, and communication with health providers as compared to rural women [26, 27].

However, the recent finding was higher than the finding reported in a study conducted in the West Shewa zone (35.8%) [19]. The reason might be due to the difference in the study setting and study participants; the current study was conducted among clients who received delivery care from general and district hospitals that provide comprehensive delivery services. Whereas, a study done in the West Shewa zone includes clients who received care in Health centers and hospitals; due to their limited scope health centers might not provide comprehensive respectful care as that of hospitals. A nationally based survey in Ethiopia revealed that health centers have a lower quality of maternal and newborn health care in all facility-level program components (input, process, and output) compared to hospitals; the overall mean quality score in input, process, and output for health centers was 49, 41, and 46 respectively, whereas the equivalent mean score for hospitals was 79, 58, and 62 [28].

In this study, the lowest score of PCMC was recorded in communication and autonomy; this was comparable with a study done in low and middle-income countries [4]. Although communication can affect the attitude of the client positively and can increase clients’ level of service satisfaction, most health professionals don’t give enough emphasis on client-provider interaction and the role of client’s autonomous decisions in their care [29].

In this study, living in rural residences had a negative association with maternal PCMC score as compared with those who reside in urban areas. This result is supported by the studies done in Addis Ababa, Bahir Dar, and Dessie [6, 7, 17]. The reason might be urban women would have better health information, facility visit experience, and client-provider interaction in their care as compared to their rural counterparts. The other explanation might also be the hospital environment’s friendliness for women; the hospital environment is near to urban women and more friendly for them compared to rural women.

The current study revealed that three up to four health providers present during labor and delivery had decreased person-centered maternity care scores as compared to one up to two health providers present during labor and delivery. This result is supported by the studies done in the North Shewa zone, Oromia region, Ethiopia, and central Ethiopia [19, 30]. This might be due to mothers not wanting to show their private bodies to more providers. The other plausible reason might also be facing challenges of communication; client-provider communication is better understood with a small number of health professionals [31].

Mothers who had no previous histories of facility delivery had a lower PCMC score as compared to their counterparts. This study is comparable to a study done in central Ethiopia [30]. This might be a result of women’s experience in their care; women who had a previous history of facility delivery will experience better communication with care providers and actively participate in their process of health care. The other reason might also if women have experience with facility delivery, the hospital environment might not be new for them; they would consider it as their second home.

Mothers who stayed at hospitals for two days had a positive association with the PCMC score as compared to mothers who stayed at hospitals only for one day. This result is comparable with a study conducted in Northeastern Ethiopia and North Shewa zone, Oromia region, Ethiopia [6, 19]. The reason might be due to as the length of stay increases the probability of experiencing the PCMC approach to care increases. This might be the result of when women stay at the hospital the women’s level of understanding about the health care system and their autonomy decision making in their care might also be increased. Evidence suggests that PCMC emphasizes the quality of the patient experience by assisting the woman to feel safe and at ease when communicating her wants and feelings to healthcare professionals [21].

In this study, mothers with the highest wealth index had increased PCMC scores as compared to those who had the lowest wealth index. This result is consistent with the studies conducted in Kenya [4]. This might be due to the reason that most respondents who had the highest wealth had a better level of education and information about their healthcare process. Besides, most of those women are living in the Urban setting that has better health information access and health care service use experience compared to the rural mothers. Moreover, women who had high incomes had better confidence and experience or were better able to get services from health institutions through good communication with healthcare providers than women with low incomes [32].

This study has some limitations; social desirability bias may be there; participants may fear disclosing their negative experience during childbirth because of thinking that the service they seek may be affected if they come again to that health institution. Besides, women’s perception of disrespected service might overestimate PCMC score; some women might not consider it as disrespectful care unless they were kicked and/or slapped.

## Conclusion

The mean score of person-centered maternity care among women who give birth in public hospitals of the South Gondar Zone was low compared with studies done in low- and middle-income countries. The highest wealth, three up to four health providers present during labor and delivery, had no previous histories of facility delivery, and two days length of stay at hospitals were factors significantly associated with person-centered maternity care. Therefore, to improve PCMC strengthening the financial empowerment of women, facility delivery, and post-delivery hospital stays for more than 24 hours is paramount. In addition, working in teamwork during labor and delivery should be encouraged.

## Supporting information

S1 Fig Schematic presentation of the sampling procedure for assessing person-centered maternity care and associated factors among women who give birth at public hospitals in South Gondar zone, North West Ethiopia, 2023. (TIF)

S1 File All necessary data are incorporated in the manuscript. The SPSS data files are freely available upon request of the corresponding author.
(TIF)

## Abbreviations

ANC: Antenatal Care
CI: Confidence Interval
PCMC: Person-Centered Maternity Care
SPSS: Statistical Package for Social Science
WHO: World Health Organization
