# Supplementary figures and images for: Person-centered maternity care and associated factors among women who give birth at public hospitals in South Gondar zone, North West Ethiopia, 2023
Source: PLoS One. 2024 Aug 22;19(8):e0303389. doi: 10.1371/journal.pone.0303389 (PMC11340964; doi:10.1371/journal.pone.0303389)

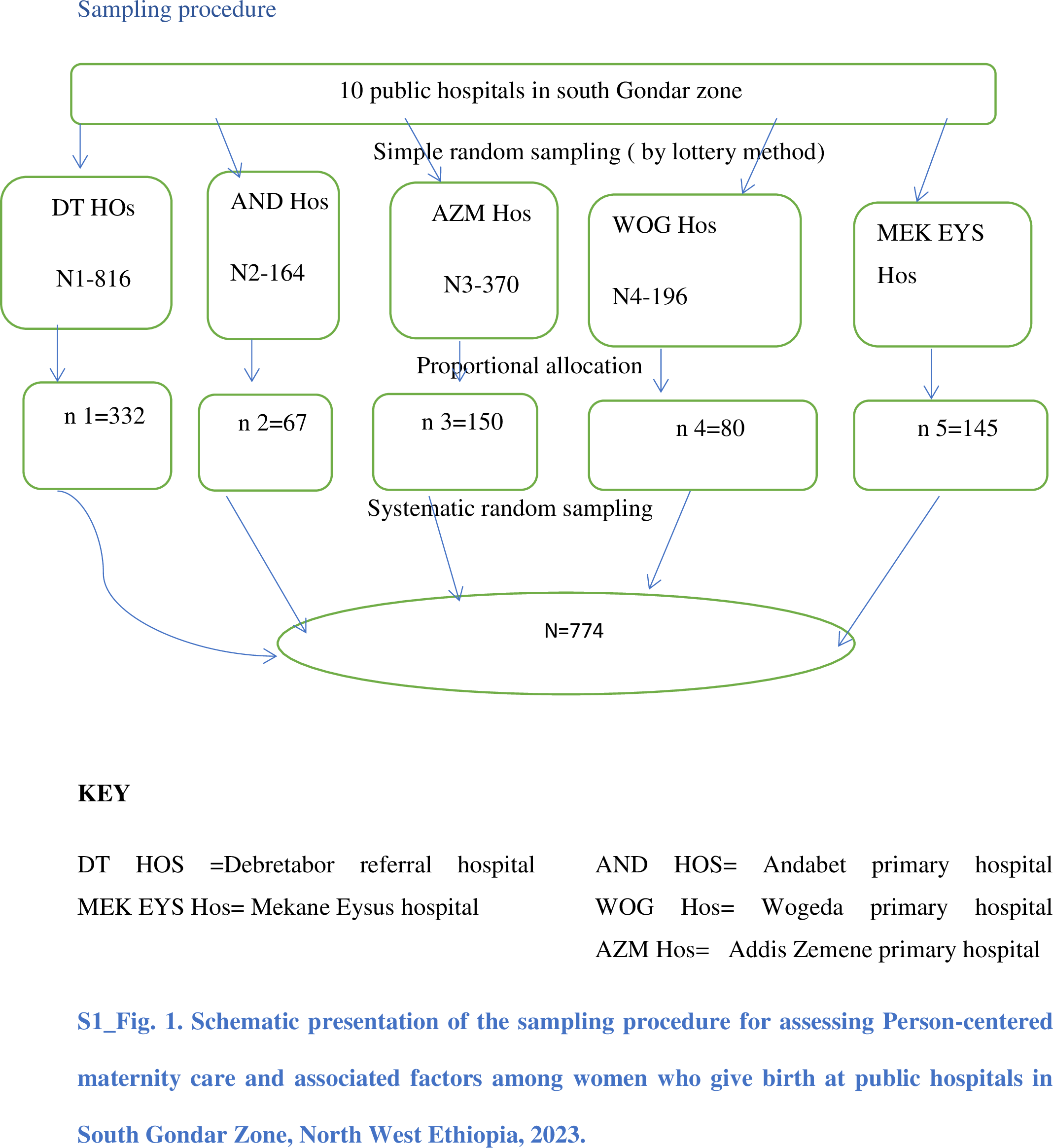

Supplement: S1 Fig — (TIF) [file pone.0303389.s001.tif]

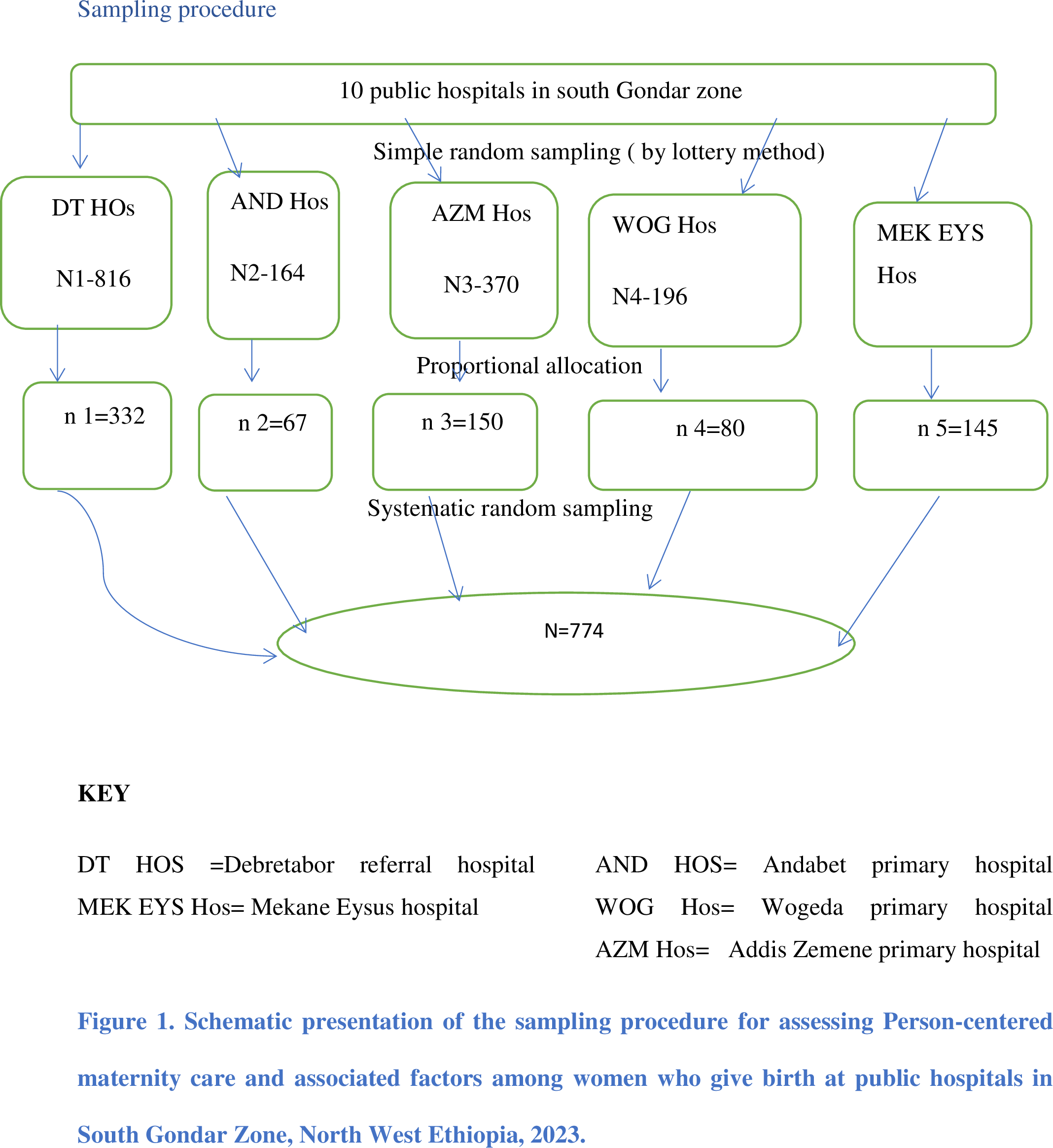

Supplement: S1 File — The SPSS data files are freely available upon request of the corresponding author. (TIF) [file pone.0303389.s002.tif]
